# Late-pregnancy uterine artery ligation increases susceptibility to postnatal Western diet-induced fat accumulation in adult female offspring

**DOI:** 10.1038/s41598-020-63392-y

**Published:** 2020-04-24

**Authors:** Forough Jahandideh, Stephane L. Bourque, Edward A. Armstrong, Stephana J. Cherak, Sareh Panahi, Kimberly F. Macala, Sandra T. Davidge, Jerome Y. Yager

**Affiliations:** 1 Department of Anesthesiology & Pain Medicine, University of Alberta, Edmonton, Alberta Canada; 2 Women and Children’s Health Research Institute, University of Alberta, Edmonton, Alberta Canada; 3 Department of Pediatrics, University of Alberta, Edmonton, Alberta Canada; 4 Department of Surgery, University of Alberta, Edmonton, Alberta Canada; 5 Department of Obstetrics and Gynecology, University of Alberta, Edmonton, Alberta Canada

**Keywords:** Intrauterine growth, Reproductive disorders

## Abstract

Stressors during the fetal and postnatal period affect the growth and developmental trajectories of offspring, causing lasting effects on physiologic regulatory systems. Here, we tested whether reduced uterine artery blood flow in late pregnancy would alter body composition in the offspring, and whether feeding offspring a western diet (WD) would aggravate these programming effects. Pregnant rats underwent bilateral uterine artery ligation (BUAL) or sham surgery on gestational day (GD)18 (term = GD22). At weaning, offspring from each group received either a normal diet (ND) or a WD. BUAL surgery increased fetal loss and caused offspring growth restriction, albeit body weights were no longer different at weaning, suggesting postnatal catch-up growth. BUAL did not affect body weight gain, fat accumulation, or plasma lipid profile in adult male offspring. In contrast, while ND-fed females from BUAL group were smaller and leaner than their sham-littermates, WD consumption resulted in excess weight gain, fat accumulation, and visceral adiposity. Moreover, WD increased plasma triglycerides and cholesterol in the BUAL-treated female offspring without any effect on sham littermates. These results demonstrate that reduced uterine artery blood flow during late pregnancy in rodents can impact body composition in the offspring in a sex-dependent manner, and these effects may be exacerbated by postnatal chronic WD consumption.

## Introduction

The last few decades have seen a global rise in the incidence and prevalence of obesity. The World Health Organization estimates that obesity affects 650 million people worldwide, with an additional 1.9 billion classified as overweight^1^. Although this trend is attributed, at least in part, to the consumption of energy-rich diets and increasingly sedentary lifestyles, a newly defined contributor to the obesity epidemic is the programming effects caused by stressors during development. The developmental origins of health and disease (DOHaD) theory posits that stressors during the fetal and immediate postnatal period can alter the growth and developmental trajectories of offspring, causing lasting effects on a number of physiologic regulatory systems. Stressors such as hypoxia, anemia, and nutrient imbalances can confer lasting metabolic dysfunction in the offspring, characterized by a propensity for excess fat accumulation^2^, as well as altered glucose metabolism and dyslipidemia^3^.

Our group has previously characterized a rat model of bilateral uterine artery ligation (BUAL) to reduce uterine artery blood flow, causing antepartum fetal ischemia, growth restriction, and neurodevelopmental outcomes broadly characteristic of cerebral palsy and developmental disability^4,5^. The BUAL surgery is performed at gestational day (GD) 18 (term = GD22), and therefore, constitutes a comparatively narrow exposure period of fetal distress. As such, this model presents a unique opportunity to study the importance of late gestation in dictating offspring body composition, which has not been studied in this model of developmental programming.

In general, many developmental outcomes are subtle, but persist indefinitely and underlying dysfunction can be exacerbated by the imposition of additional stressors (e.g. advanced age, consumption of a high fat diet)—the so called ‘two-hit hypothesis’. For example, growth restricted offspring born to pregnancies complicated by prenatal hypoxia, anemia, and/or protein restriction exhibit metabolic dysfunction that is exacerbated by postnatal feeding of a high-fat and high-sucrose diet^6,7^. Moreover, sexual dimorphism in programming outcomes have been extensively documented, with male and female offspring exhibiting different susceptibilities to long-term health complications^8^. Although the long-term effects of BUAL on various cardio metabolic health outcomes in the offspring have been reported to be sex-dependent^9^, the effects on body composition have not been reported.

The purpose of the present study was to investigate whether BUAL during late pregnancy alters the developmental trajectories in male and female offspring, thereby affecting long-term metabolic function. We also sought to determine whether chronic consumption of a high-fat and high-sucrose diet, termed Western Diet (WD) herein, in offspring would exacerbate these programming effects. Indeed, the WD, mimicking a typical North American diet constitutes a metabolic stress that is relevant to populations in both advanced and developing countries with a rapid transition to more obesogenic foods^10,11^.

## Results

**Figure 1:**
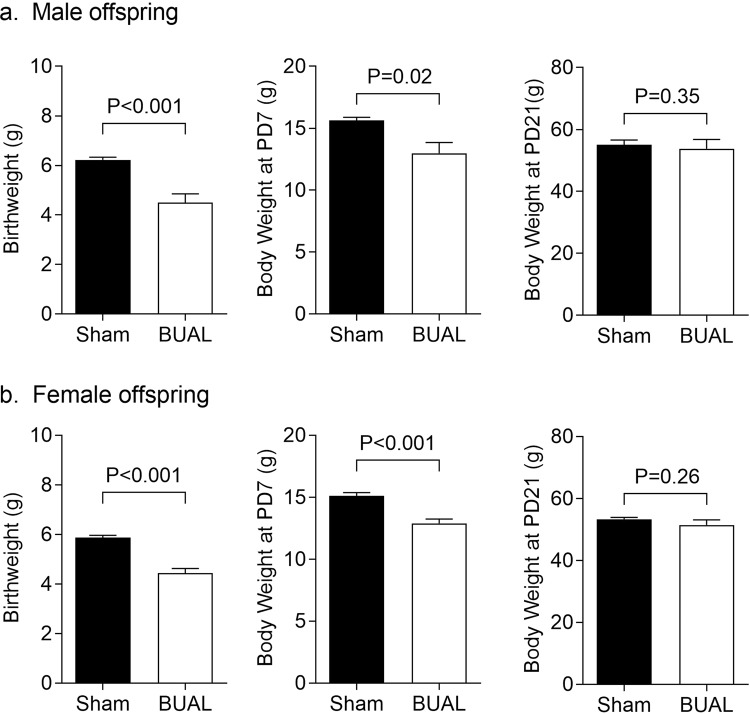
Offspring body weights at postnatal days (PD) 1, 7, and 21 in (**a**) male; and (**b**) female offspring born to dams that underwent sham surgery or bilateral uterine artery ligation (BUAL) from gestational day 18 to 22. Data presented as Mean ± SEM for N = 7–8 offspring from separate litters in each group and analyzed by student’s *t*-test.

BUAL had a profound impact on pregnancy outcomes. Dams that underwent the BUAL had smaller litter sizes at birth (Sham: 13.9 ± 1.0, n = 7; BUAL: 6.6 ± 0.1, n = 7; P < 0.001) but sex-distribution of litters was not affected (Sham: 47 ± 5% males, n = 7; BUAL: 45 ± 4% males, n = 7; P = 0.74). BUAL caused 42% reduction in body weight in male offspring (P < 0.001, Fig. 1a), and a 34% reduction body weight in female offspring (P < 0.001; Fig. 1b). Despite the profound growth restriction caused by BUAL, both male and female offspring exhibited catch-up growth in the immediate postnatal phase, such that neither male nor female offspring body weights were different by PD21 (Fig. 1).

When assessing postnatal food intake, we observed a sharp decrease in food intake over time in the offspring (Supplementary Fig. S1). However, BUAL did not affect food intake in male or female offspring as shown by the area under the food intake curve (Supplementary Fig. S1).

**Figure 2:**
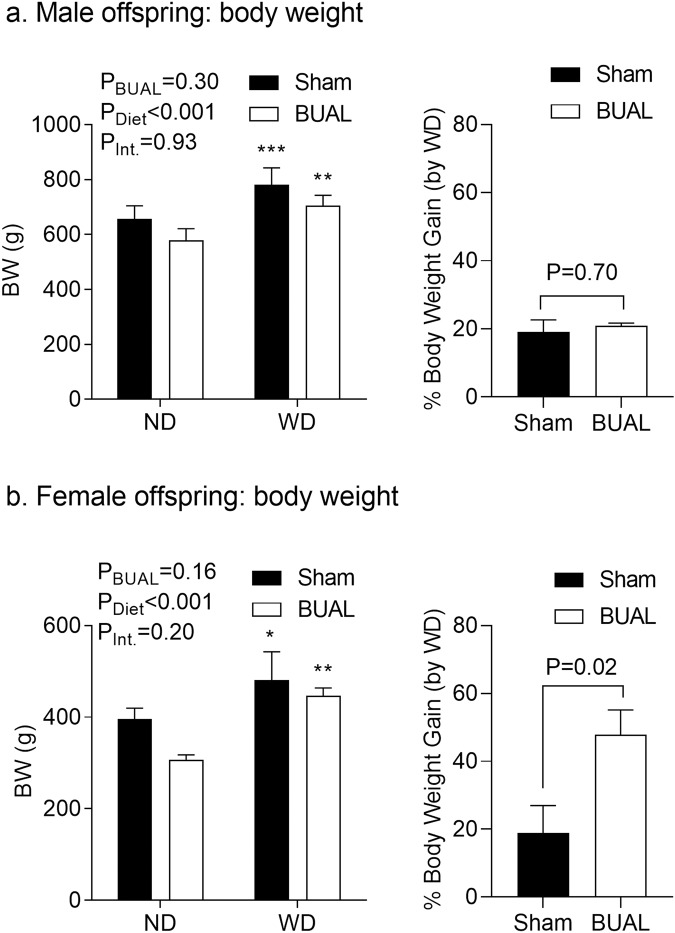
Body weights and %body weight gain by Western diet (WD) in adult male (**a**); and female (**b**) offspring at ~24 wks of age. Data are presented as Mean ± SEM for N = 4–7 offspring from separate litters in each group. Body weights data were analyzed by two-way ANOVA followed by Holm-Sidak *post-hoc* comparison test. Body weight gain data were analyzed by student’s *t*-test. *p < 0.05. *P < 0.05, **P < 0.01, and ***P < 0.001 compared to normal diet (ND)-fed littermates.

Feeding offspring a calorically-dense WD caused a BW gain in both adult male and female offspring compared to ND-fed littermates (Fig. 2). In male offspring, the effect of WD in BW gain was similar in both control and BUAL groups (Fig. 2a). In female offspring, the effects of WD were more pronounced in BUAL offspring, such that when comparing WD-fed offspring relative to their ND-fed littermates, BUAL female offspring had greater proportional weight gain compared to sham offspring (P = 0.02; Fig. 2b).

BUAL did not affect relative organ weights in either male or female offspring (Table 1). However, there was an overall effect of postnatal diet in male and female offspring. In male offspring, heart and spleen weights were larger in WD-fed offspring, but these differences did not persist when organ weights were normalized to body weight. In females, heart, kidney, and spleen weights were larger in WD-fed offspring, and when normalized to body weight, heart and kidney weights were relatively smaller compared to ND-fed offspring.

**Table 1:** Effects of maternal bilateral uterine artery ligation (BUAL) surgery on organ weights of male and female offspring fed a normal diet (ND) or western diet (WD) chronically.

Organ	Weight	ND		WD		P value		
		Sham	BUAL	Sham	BUAL	BUAL	Diet	Interaction
**Male Offspring**								
Liver	Absolute (g)	19.61 ± 1.78	17.99 ± 2.12	22.92 ± 2.83	19.92 ± 1.46	0.484	0.127	0.501
	Relative (%BW)	3.00 ± 0.18	3.08 ± 0.19	2.92 ± 0.21	2.82 ± 0.13	0.928	0.148	0.370
Heart	Absolute (g)	1.29 ± 0.04	1.28 ± 0.10	1.43 ± 0.09	1.70 ± 0.19^**†**^	0.342	**0.011**	0.132
	Relative (%BW)	0.20 ± 0.02	0.22 ± 0.01	0.19 ± 0.02	0.24 ± 0.02	0.130	0.900	0.162
Kidneys	Absolute (g)	2.69 ± 0.11	2.59 ± 0.37	3.10 ± 0.35	3.00 ± 0.14	0.699	0.141	0.991
	Relative (%BW)	0.37 ± 0.02	0.46 ± 0.08	0.36 ± 0.02	0.43 ± 0.04	0.164	0.649	0.862
Spleen	Absolute (g)	0.79 ± 0.05	0.78 ± 0.05	0.82 ± 0.03	0.96 ± 0.07	0.276	**0.037**	0.164
	Relative (%BW)	0.12 ± 0.02	0.14 ± 0.01	0.11 ± 0.01	0.14 ± 0.01	0.188	0.144	0.393
**Female Offspring**								
Liver	Absolute (g)	10.48 ± 0.68	9.28 ± 0.85	13.99 ± 4.08	10.92 ± 0.78	0.316	0.228	0.656
	Relative (%BW)	2.73 ± 0.20	3.02 ± 0.22	2.72 ± 0.38	2.45 ± 0.17	0.486	0.146	0.379
Heart	Absolute (g)	0.84 ± 0.16	0.82 ± 0.10	0.97 ± 0.24	1.05 ± 0.10^**†**^	0.690	**0.010**	0.337
	Relative (%BW)	0.22 ± 0.07	0.27 ± 0.02	0.21 ± 0.06	0.24 ± 0.03^**†**^	0.184	**P** < **0.001**	0.485
Kidneys	Absolute (g)	1.82 ± 0.16	1.76 ± 0.10	2.22 ± 0.30	2.18 ± 0.06	0.910	**0.021**	0.822
	Relative (%BW)	0.44 ± 0.04	0.49 ± 0.05	0.38 ± 0.04	0.42 ± 0.05^**†**^	0.435	**0.023**	0.214
Spleen	Absolute (g)	0.46 ± 0.07	0.49 ± 0.04	0.54 ± 0.05	0.66 ± 0.06	0.207	**0.045**	0.418
	Relative (%BW)	0.12 ± 0.02	0.16 ± 0.01	0.12 ± 0.01	0.15 ± 0.02	0.089	0.474	0.910

^†^P < 0.05 compared ND and WD in the same surgery group.

**Figure 3:**
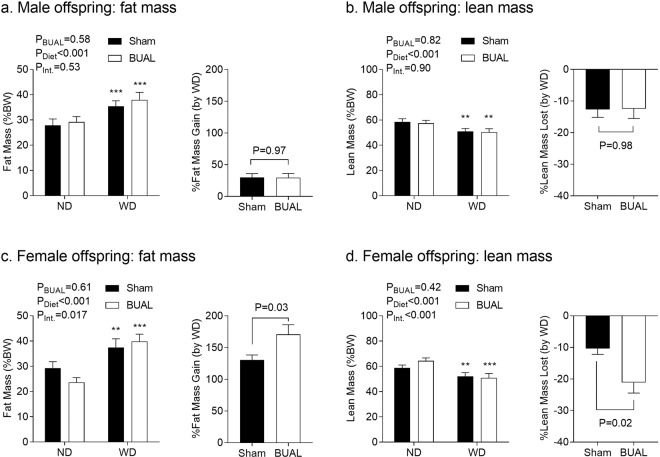
Body composition, including (**a**,**c**) fat mass; and (**b**,**d**) lean mass, in adult offspring at ~24 wks of age, as assessed by ECHO-MRI. Data are presented as Mean ± SEM for N = 6–7 offspring from separate litters in each group. Fat and lean mass data were analyzed by two-way ANOVA followed by Holm-Sidak *post-hoc* comparison test. Fat and lean mass gain data were analyzed by student’s *t*-test. **P < 0.01 and ***P < 0.001 compared to ND-fed littermates.

We next assessed changes in body composition in adult offspring (Fig. 3). As expected, consumption of a WD led to increases in whole body fat content in male (P < 0.001; Fig. 3a) and female offspring (P < 0.001; Fig. 3c), and a corresponding opposite trend for lean body mass (P < 0.001 for both sexes; Fig. 3b,d). In male offspring, there was no effect of BUAL on body composition. In female offspring, there was interaction between BUAL and postnatal diet in both fat mass (P = 0.017) and lean mass (P < 0.001), with BUAL WD-fed offspring exhibiting proportionally more fat (P = 0.03; Fig. 3c) and less lean mass (P = 0.02; Fig. 3d) relative to their ND-fed littermates compared to sham offspring.

**Figure 4:**
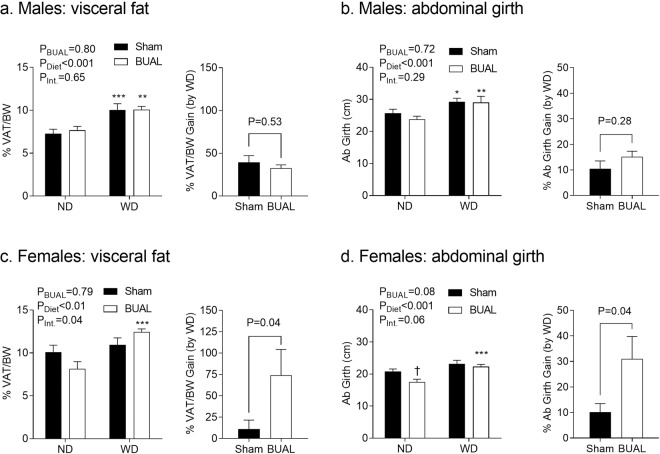
Indices of abdominal obesity, including (**a**) combined weight of fat pads including perirenal, epididymal, mesenteric, and omental fat pads (normalized to body weight); (**c**) combined weight of fat pads including perirenal, mesenteric, and omental fat pads (normalized to body weight); and (**b**,**d**) abdominal circumference in adult offspring at ~24 wks of age. Data are presented as Mean ± SEM for N = 4–7 offspring from separate litters in each group. Data were analyzed by two-way ANOVA followed by Holm-Sidak *post-hoc* comparison test. Data on visceral fat and abdominal girth gain (by WD) were analyzed by student’s *t*-test or Mann-Whitney test. *P < 0.05, **P < 0.01, and ***P < 0.001 compared to ND-fed littermates. ^**†**^P < 0.05 compares sham and BUAL in same diet groups.

Because total fat content may reflect both subcutaneous and visceral depots, we measured visceral fat content by collecting and weighing the intra-abdominal fat depots (see methods), as well as measuring abdominal girth (as an index of abdominal fat^12^) in male and female offspring. In both males and females, post-weaning WD feeding increased visceral fat accumulation (P < 0.001 for both sexes; Fig. 4a,c) and abdominal girth (P < 0.001 for both sexes; Fig. 4b,d). In male offspring, there was no effect of BUAL on either visceral fat (Fig. 4a) or abdominal girth (Fig. 4b). Analysis of fat depots including retroperitoneal, epididymal, mesenteric, and omental fat pads also revealed no effect of BUAL on these parameters in male offspring (Table 2).

**Table 2:** Effects of maternal bilateral uterine artery ligation (BUAL) surgery on fat depots of male and female offspring fed a normal diet (ND) or western diet (WD) chronically.

Organ	Weight	ND		WD		P value		
		Sham	BUAL	Sham	BUAL	BUAL	Diet	Interaction
**Male Offspring**								
Retroperitoneal fat	Absolute (g)	24.14 ± 2.88	21.10 ± 2.52	43.02 ± 5.82^9^	36.17 ± 3.09^**‡**^	0.382	**0.0001**	0.436
	Relative (%BW)	3.61 ± 0.19	3.61 ± 0.22	5.43 ± 0.46^9^	5.09 ± 0.24^**‡**^	0.685	**0.0002**	0.553
Epididymal fat	Absolute (g)	11.01 ± 1.84	10.06 ± 1.22	17.05 ± 2.80^**†**^	17.44 ± 2.18^**‡**^	0.844	**0.0008**	0.488
	Relative (%BW)	1.62 ± 0.18	1.71 ± 0.10	2.18 ± 0.31	2.47 ± 0.25^**†**^	0.595	**0.006**	0.505
Mesenteric fat	Absolute (g)	11.25 ± 1.55	10.94 ± 1.27	14.79 ± 1.22^**‡**^	13.92 ± 1.59^**†**^	0.826	**0.001**	0.503
	Relative (%BW)	1.67 ± 0.13	1.87 ± 0.13	1.90 ± 0.10	1.95 ± 0.13	0.374	0.186	0.454
Omental fat	Absolute (g)	2.54 ± 0.41	2.85 ± 0.72	4.23 ± 0.61^**‡**^	3.94 ± 0.50^**†**^	0.980	**0.0005**	0.238
	Relative (%BW)	0.37 ± 0.04	0.47 ± 0.10	0.53 ± 0.06^**†**^	0.55 ± 0.04	0.445	**0.021**	0.366
**Female Offspring**								
Retroperitoneal fat	Absolute (g)	30.79 ± 4.23	19.78 ± 2.62	39.30 ± 4.23^**†**^	40.34 ± 2.06^9^	0.326	**P** < **0.001**	**0.009**
	Relative (%BW)	7.58 ± 0.66	6.34 ± 0.69	8.34 ± 0.73	9.02 ± 0.30^**†**^	0.7156	**0.013**	0.116
Mesenteric fat	Absolute (g)	7.76 ± 1.22	4.38 ± 0.76*	9.56 ± 1.01	11.61 ± 0.71^9^	0.638	**P** < **0.001**	**0.002**
	Relative (%BW)	1.90 ± 0.18	1.40 ± 0.21	2.04 ± 0.19	2.61 ± 0.17^9^	0.843	**0.003**	**0.010**
Omental fat	Absolute (g)	2.44 ± 0.32	1.23 ± 0.12*	2.74 ± 0.49	3.50 ± 0.54^9^	0.669	**0.001**	**0.004**
	Relative (%BW)	0.60 ± 0.05	0.40 ± 0.04	0.57 ± 0.09	0.79 ± 0.12^**‡**^	0.894	**0.017**	**0.005**

*P ≤ 0.05 compares sham and BUAL in same diet group. ^**†**^P < 0.05, ^**‡**^P < 0.01, ^9^P < 0.001 compares ND and WD in the same surgery group.

In female offspring, an interaction between BUAL and postnatal diet was observed in total visceral fat mass (P = 0.04; Fig. 4c), as well as trend in abdominal girth (P = 0.06; Fig. 4d). When WD-fed offspring outcomes were calculated as a relative change compared to their ND-fed littermates, BUAL female offspring had higher visceral fat mass (P = 0.04; Fig. 4c) and abdominal girth (P = 0.04; Fig. 4d), consistent with BW gain and body composition parameters. When comparing individual depots, an interaction between BUAL and postnatal diet was observed in absolute weights of mesenteric and omental fat pads, and all fat pads when normalized to body weight (Table 2).

**Figure 5:**
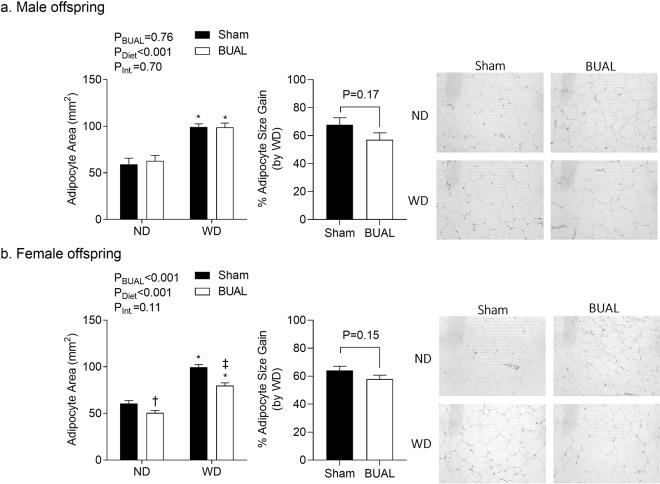
Adipocyte size in (**a**) adult male; and (**b**) adult female offspring at ~24 weeks of age. Left panels show summarized data and right panels show representative images. Data are presented as Mean ± SEM for N = 5–7 offspring from separate litters in each group. Data on adipocyte area were analyzed by two-way ANOVA followed by Holm-Sidak *post-hoc* comparison test. Data on adipocyte size gain (by WD) were analyzed by student’s *t*-test. *P < 0.05 compares to ND-fed littermates. ^**†**^P < 0.05, ^**‡**^P < 0.01 compares sham and BUAL in the same diet group.

We next examined adipocyte size in adult offspring. WD caused an increase in adipocyte size in male (P < 0.001; Fig. 5a) and female offspring (P < 0.001; Fig. 5b). In male offspring, there was no effect of BUAL on adipocyte size as shown in Fig. 5a. Interestingly, in female offspring, adipocyte size was reduced by BUAL (Fig. 5b), and no interaction was observed.

**Figure 6:**
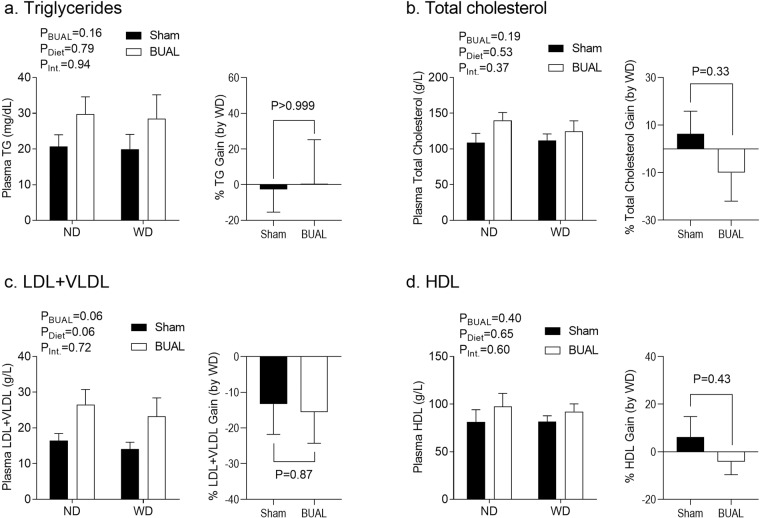
Indices of plasma lipid profile, including (**a**) triglyceride; (**b**) total cholesterol; (**c**) low density lipoprotein and very low density lipoprotein cholesterol; and (**d**) high density lipoprotein cholesterol in adult male offspring at ~24 wks of age. Data are presented as Mean ± SEM for N = 3–5 offspring from separate litters in each group. Data on lipid components were analyzed by two-way ANOVA. Data on % gain (by WD) were analyzed by student’s *t*-test.

**Figure 7:**
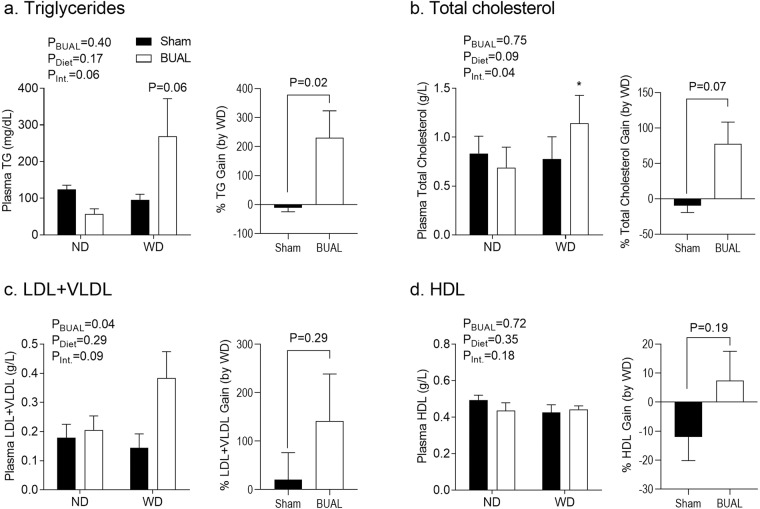
Indices of plasma lipid profile, including (**a**) serum triglyceride; (**b**) total cholesterol; (**c**) low density lipoprotein and very low density lipoprotein cholesterol; and (**d**) high density lipoprotein cholesterol in adult female offspring at ~24 wks of age. Data are presented as Mean ± SEM for N = 3–5 offspring from separate litters in each group. Data on lipid components were analyzed by two-way ANOVA followed by Holm-Sidak *post-hoc* comparison test. Data on % gain (by WD) were analyzed by student’s *t*-test. *P < 0.05 compared to ND-fed littermates.

Next, we tested plasma lipid profile in the offspring. There was no overall effect of post-weaning diet on plasma lipids in either sex (Figs. 6 & 7). While there was no overall effect of BUAL on lipid profile in the male offspring (Fig. 6), WD-fed females in the BUAL group tended to have increased plasma TG levels compared to their ND-fed littermates. BUAL WD-fed offspring exhibited proportionally higher plasma TG (P = 0.02; Fig. 7a) relative to ND-fed littermates compared to sham offspring. A similar but more robust pattern was seen in total plasma cholesterol levels (Fig. 7b); WD-fed offspring in the BUAL group had higher plasma total cholesterol levels compared to their ND-fed littermates. Separation of cholesterol fractions revealed a similar pattern in LDL + VLDL levels (Fig. 7c), whereas plasma HDL levels were not affected by prenatal treatment (Fig. 7d).

**Figure 8:**
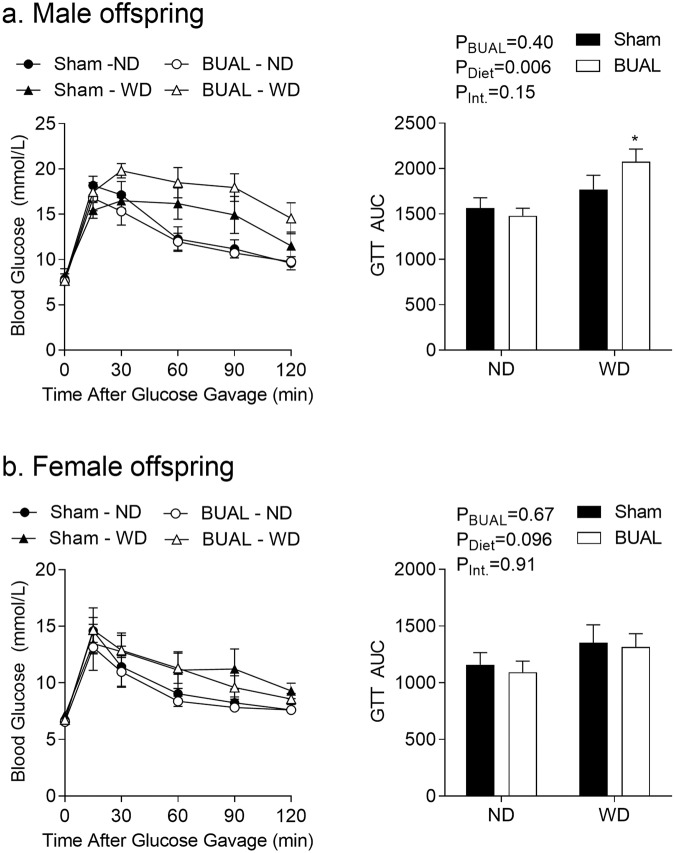
Glucose tolerance tests (GTT) in adult (**a**) male; and (**b**) female offspring at ~24 weeks of age. Data are shown as the time course and summarized as area under the curve (AUC). Data are presented as Mean ± SEM for N = 5–7 offspring from separate litters in each group and analyzed by two-way ANOVA followed by Holm-Sidak *post-hoc* comparison test. *P < 0.05 compared to ND-fed littermates.

Finally, we assessed glucose handling in the offspring. There were no effects of either prenatal treatment or post-weaning diet on fasting glucose levels in either sex (data not shown). In male offspring, WD consumption was associated with altered glucose handing, as indicated by an increase in area under the GTT curve (AUC, P = 0.006, Fig. 8a), albeit only BUAL offspring fed the WD exhibited increased AUC compared to the ND-fed littermates. Interestingly, in female offspring, neither WD nor BUAL was associated with altered glucose handling (Fig. 8b).

## Discussion

Intrauterine growth restriction is a common pregnancy complication especially in developing countries; the incidence of intrauterine growth restriction is 6-fold higher in such countries compared to developed countries^13^. The BUAL rat model, where uterine artery blood flow is restricted during the last few days of pregnancy constitutes a comparatively severe, albeit short-term insult, and has been shown to recapitulate many of the features of asymmetric growth restriction^14,15^, which characterizes the majority of cases of intrauterine growth restriction^16^. Here, we used this model to investigate the impact of this fetal stress on body composition and metabolic function in the offspring. We have previously shown that this prenatal intervention causes altered postnatal growth trajectories and a number of neurodevelopmental deficits that persist into adulthood^17^. Here, our results show that the late phase of gestation in the rat is important in dictating body composition in addition to metabolic function^9^, and these effects are influenced by the sex of the offspring. Although the maternal BUAL model is not a fetal nutrient restriction or fetal ischemic insult per se, the findings of the current study support previous studies showing fetal stressors such as hypoxia^6^, iron deficiency anemia^7^, and macronutrient perturbations^18^ can impact long-term metabolic programming in the offspring. Notably, the BUAL model is distinct from most other developmental programming models in that the insult occurs during a comparatively narrow window (the last four days of pregnancy), thus implicating this period of gestation as an important determinant of body composition in adulthood.

We observed marked sex-based differences in susceptibility of offspring to the programming of body composition and metabolic function. Indeed, despite having a marked impact on offspring survival rates and causing intrauterine growth restriction in both male and female offspring, we found that BUAL had no observable long-term impact on body composition or the accumulation of abdominal fat in adult male offspring. In contrast, female offspring, exposed to the same BUAL-induced stress, exhibited marked differences from sham offspring, depending on the postnatal diet they consumed. BUAL-exposed female offspring fed the normal diet post-weaning tended to be smaller, and have a greater amount of lean mass, and a correspondingly lower quantity of fat, both when measured as total body fat content and visceral adipose tissue. Several studies have shown clear differences in the susceptibility of offspring to the programming effects of a wide range of prenatal stressors, such that male and female offspring exhibit qualitatively different responses in the nature, timing and severity of these programming outcomes^19–23^. The present results are interesting because studies using the BUAL model report greater susceptibility of male offspring to the long-term programming of cardiovascular and metabolic function (reviewed in^9^), although a few studies have reported more pronounced outcomes in females compared to their male counterparts^20,21^.

The lack of altered body composition in the female offspring fed the ND underscores the value of imposing secondary stressors to study underlying dysfunction. Nevertheless, the lack of clear metabolic dysfunction in the female BUAL-ND offspring compared to their sham littermates, and even improved outcomes in some parameters (e.g. absolute mesenteric and omental fat depots, abdominal girth, and adipocyte area) was unexpected, particularly within the context of the predictive adaptive responses or thrifty phenotype theories, which would predict a greater propensity for energy storage (and hence fat accumulation). Although this may reflect an overall healthier metabolic state, adiposity in females provides the metabolic means of producing energetically costly offspring^24^. Therefore, the long-term implications of altered body composition on reproductive function remain an important question for future studies. In contrast, when given a diet high in sucrose and fat, programmed female offspring exhibited increased proportional fat accumulation and reduced lean muscle mass accumulation. In principle, the exaggerated weight gain and body fat accumulation is consistent with the predictive adaptive theory^25^, wherein the mismatch between nutrient availability between the developmental (prenatal period) and postnatal periods results in an excess accumulation of fat, because the offspring adopts a phenotype adept at conserving energy (i.e. obesity). Alternatively, the nature of the prenatal stress in the present study (severe, immediate onset, and of relatively short duration) may be such that the long-term programming effects reflect a direct consequence of the ischemia on fetal or placental growth, rather than a concerted attempt by the fetuses to predict and adapt to a forthcoming environment. For example, sex-based differences have been reported in placental and fetal growth trajectories in rats, such that female offspring exhibit reduced placental labyrinth, placental blood space volume, as well as fetal blood space surface area compared to males during the last week of gestation^25^; this lag in placental development coincided with reduced growth in female fetuses^26^. Because the placenta is the gestational interface between the mother and fetus, its adaptive properties are essential for optimal fetal development, and a reduced exchange capacity may cause female fetuses to be particularly susceptible to severe insults during this latter stage of gestation.

The evidence linking uteroplacental ischemia and glucose intolerance in adult offspring has been inconsistent among various studies. Siebel *et al*. reported male offspring, but not female offspring, had altered glucose handling in adulthood^27^. However, it should be noted that male offspring in this study also exhibited increased adiposity, whereas female did not. In contrast, Jansson *et al*.^20^, using a similar model, reported that female offspring developed altered glucose handling (and other metabolic alterations), and male offspring did not. Given that a similar model was used in these studies, it is difficult to reconcile these apparent discrepancies. Here, although male BUAL offspring exhibited no changes in body composition or plasma lipid profile, even after prolonged feeding of a WD, these offspring showed early signs of altered glucose handling, as indicated by an increased AUC in the GTT curve. A notable difference in the present study is that altered glucose handling was only evident when offspring were fed the WD, further complicating the interpretation of the results. These differences may underscore the importance of postnatal environmental factors including those inherent to different strains (e.g. quality of maternal care and size of litter) in dictating long-term metabolic dysfunction.

Interestingly, despite a clear difference in body composition and visceral adipose tissue accumulation between ND and WD-fed female offspring in the BUAL group, we observed no obvious alterations in glucose handling. This outcome was unexpected, given that excess visceral fat is an important contributor to chronic disease outcomes^28^. However, at the least in the present study, the altered glucose handling cannot be attributed to excess fat deposition per se. Moreover, while quantity of visceral fat is important, adipocyte morphology and function are also essential determinants of metabolic function. Larger adipocytes as associated with increased inflammatory phenotypes and reactive oxygen species production are deemed to be important in chronic inflammation and release of adipokines that contribute to the progression of metabolic disease^29^. Female offspring in the BUAL group has smaller adipocytes, which may mitigate the metabolic consequences of the excess fat deposition. It is also possible that more overt metabolic changes such as glucose intolerance and insulin resistance may develop as the offspring age; indeed, at 6 months of age, rats are relatively young and are thought to correspond to human ages of less 20 years^30^. The role of altered beta cell morphology^31^ and the interaction with sex hormones in dictating the metabolic phenotype also warrants further investigation^32,33^. Notwithstanding the lack of glucose handling in females, the elevated lipid profiles in WD-fed BUAL offspring suggests metabolic dysfunction that may culminate into overt disease over time.

The present findings demonstrate that adult female offspring are vulnerable to the programming effects of reduced uterine artery blood flow in the last four days of pregnancy. The mechanism underlying these metabolic programming effects are presently unclear, but could involve myriad systems and signaling pathways, ranging from appetite regulation to physical activity, to endocrine signaling, all of which have been shown to be plastic in the perinatal period, and hence influenced by perinatal stressors^34,35^. Notwithstanding, the results presented herein demonstrate that the programming of long-term metabolic function is dependent upon time, duration, and severity of the insult, as well as the sex of the offspring. Furthermore, an important consideration is that prenatal insults may cause long-term programming effects that are not invariably deleterious for offspring health but can be exacerbated when a second stressor is presented.

## Materials and Methods

### Animals and treatments

The experimental protocols described herein conform to the guidelines of the Canadian Council on Animal Care, and were approved by the University of Alberta Animal Care and Use Committee (Protocol No. 0000364). Twelve-week old Long-Evans female rats were purchased from Charles River Laboratories Inc. (Saint-Constant, QC) and housed in the Animal Care Facility at the University of Alberta. Environmentally, a 12:12 hr light-dark cycle (07:00–19:00) at an ambient temperature of 23 °C with 40–60% relative humidity was maintained; all rats had *ad libitum* access to food and water throughout the study. After 1 week of acclimation, females were mated with age-matched males; the onset of pregnancy (gestational day [GD]1) was confirmed by the presence of sperm in vaginal smears the following morning. Pregnant dams (N = 14) were randomized to either a sham (N = 7) or BUAL surgery (N = 7) group. The surgical intervention consisted of a bilateral uterine artery ligation (BUAL) performed on GD18 to induce placental insufficiency. Rats in the BUAL group were anesthetized with isoflurane (4% induction, 2% maintenance in air, and a midline abdominal incision (2–3 cm in length) was made. Both uterine arteries, proximal to the uterine bifurcation, were ligated with 4-0 vicryl suture (Whicon Inc. Somerville, NJ). The muscular layer was then sutured with Vicryl, treated topically with bupivicaine, and the skin layer was sutured with 5-0 silk (Angiotech Surgical Specialties Corp., Reading, PA, USA). Sham animals underwent an identical procedure, albeit the uterine arteries were not ligated. Delivery occurred vaginally on GD22.

At birth (postnatal day [PD]1), litters were reduced to 10 offspring to standardize postnatal conditions, and all dams continued to receive their standard grain-based rodent diet. At the time of weaning (PD21) all offspring were fed a purified normal diet (ND; Research Diets Inc. D10012G) or high-fat and high-sucrose Western diet (WD; Research Diets Inc. D12451) consisting of 45% energy from fat (40% derived from lard and 5% from soybean oil) and 17% energy from sucrose. Weekly body weight (BW) and food intake in the offspring was measured at postnatal weeks of 5–6, 11–12, and 18–20.

### Glucose tolerance test (GTT)

At approximately 20 weeks of age, offspring were fasted overnight, and baseline glucose levels were assessed from a saphenous venipuncture using an AccuChek Aviva Nano blood glucometer (Roche Diagnostics). Rats were then administered a glucose solution (0.5 g D-glucose per mL water) by oral gavage at a dose of 2 g/kg body weight. Blood glucose concentrations were then assessed at 15, 30, 60, 90, and 120 minutes by saphenous venipuncture following glucose administration.

### Body composition and tissue collection

Body composition was assessed at 24 weeks of age in conscious rats using a whole-body composition analyzer (EchoMRI 4-in-1/1000, Echo Medical Systems). Offspring were subsequently anesthetized with isoflurane (4% in 100% O_2_) and euthanized by exsanguination via cardiac puncture. Organs were collected, rinsed in phosphate-buffered saline (PBS) (0.1 M, pH 7.4), blotted dry, and weighed. Tissue samples were then frozen in liquid nitrogen and stored at −80 °C. Adipose tissue from visceral fat depots (omental, mesenteric, epididymal, and retroperitoneal) was collected and weighed; adipose tissue samples from the retroperitoneal depot were fixed in 4% neutral buffered formalin (pH 7.4) at 4 °C overnight, rinsed with PBS (pH 7.4), and stored in 70% EtOH at 4 °C for morphological analysis.

### Plasma lipid profile

Plasma triglycerides (TG) (Cayman Chemicals; Ann Arbor, Michigan USA), total, HDL, and LDL + VLDL cholesterol (Abcam Inc. Toronto, ON, Canada) concentrations were measured using direct colorimetric enzymatic reactions as per the manufacturer’s instructions.

### Adipocyte size and distribution

Adipose tissues were embedded in paraffin blocks, cut into 5 µm sections, and fixed on glass slides. One slide per sample was stained with hematoxylin-eosin, and 10/ photomicroscopic images of each slide were taken in a grid formation using a 20X objective lens and AxioVision 4.8 software. ImageJ software “freehand selections” tool was used to measure adipocyte area (mm^2^).

### Statistical analyses

In all cases, N values reflect the number of litters (or dams) from which offspring were obtained. In cases where multiple offspring were sampled from the same litter, mean values were calculated and considered N  =  1; this corresponds to 7 pups per N in the offspring BW at PD1,7, and 21 data set, and 1 pup for all other data sets presented. Data presented as mean ± SEM were assessed for normality by the Shapiro-Wilk test. Litter outcomes were compared by Student’s *t*-test. Offspring outcomes were analyzed by two-way ANOVA, for the main effects of prenatal intervention (sham versus BUAL) and diet (ND versus WD), followed with Holm-Sidak *post hoc* test where main effects were found. The effects of WD relative to ND-fed littermates were also analyzed either by Student’s *t*-test or Mann-Whitney test as appropriate. Histology was analyzed by two independent investigators, one of whom was blinded to the experimental conditions. All statistical analyses were conducted using Prism 8.0 (GraphPad Software, Inc.).

## Supplementary information

Supplementaryinformation
