# Supplementary material for: Late-pregnancy uterine artery ligation increases susceptibility to postnatal Western diet-induced fat accumulation in adult female offspring
Source: Sci Rep. 2020 Apr 24;10:6926. doi: 10.1038/s41598-020-63392-y (PMC7181802; doi:10.1038/s41598-020-63392-y)
Supplement: Supplementary file 1 — Supplementaryinformation [file 41598_2020_63392_MOESM1_ESM.docx]

Late-pregnancy uterine artery ligation increases susceptibility to postnatal Western diet-induced fat accumulation in adult female offspring

Forough Jahandideh^1,2,†^, Stephane L. Bourque^1,2,3,†^, Edward A. Armstrong^3^, Stephana J. Cherak^1^, Sareh Panahi^1^, Kimberly F. Macala^2,4^, Sandra T. Davidge^2,5^ and Jerome Y. Yager^2,3,*^

**Supplementary Figure S1.** Normalized weekly food intake over time in male and female offspring. N=4-6 offspring from separate litters in each group.
